# EVOAC-HP: An Efficient and Verifiable Outsourced Access Control Scheme with Hidden Policy

**DOI:** 10.3390/s23094384

**Published:** 2023-04-28

**Authors:** Haobin Ma, Dehua Zhou, Peng Li, Xiaoming Wang

**Affiliations:** College of Information Science and Technology, Jinan University, Guangzhou 510632, China; ponyhb159@stu2020.jnu.edu.cn (H.M.); pengli@jnu.edu.cn (P.L.); twxm@jnu.edu.cn (X.W.)

**Keywords:** access control, data sharing, Attribute-Based Encryption, hidden policy, outsourced decryption

## Abstract

As medical data become increasingly important in healthcare, it is crucial to have proper access control mechanisms, ensuring that sensitive data are only accessible to authorized users while maintaining privacy and security. Ciphertext-Policy Attribute-Based Encryption (CP-ABE) is an attractive access control solution that can offer effective, fine-grained and secure medical data sharing, but it has two major drawbacks: Firstly, decryption is computationally expensive for resource-limited data users, especially when the access policy has many attributes, limiting its use in large-scale data-sharing scenarios. Secondly, existing schemes are based on data users’ attributes, which can potentially reveal sensitive information about the users, especially in healthcare data sharing, where strong privacy and security are essential. To address these issues, we designed an improved CP-ABE scheme that provides efficient and verifiable outsourced access control with fully hidden policy named EVOAC-HP. In this paper, we utilize the attribute bloom filter to achieve policy hiding without revealing user privacy. For the purpose of alleviating the decryption burden for data users, we also adopt the technique of outsourced decryption to outsource the heavy computation overhead to the cloud service provider (CSP) with strong computing and storage capabilities, while the transformed ciphertext results can be verified by the data user. Finally, with rigorous security and reliable performance analysis, we demonstrate that EVOAC-HP is both practical and effective with robust privacy protection.

## 1. Introduction

The rapid advancement of medical technology has revolutionized the healthcare industry, and one of its most significant impacts is the increasing prevalence of electronic health record (EHR) systems, replacing traditional paper-based medical records [1,2]. The adoption of EHRs has facilitated the real-time management and sharing of medical records for both doctors and patients. Additionally, healthcare practitioners can now access data more easily, enabling them to make more comprehensive diagnoses. With the emergence of 5G, cloud computing and big data, cloud-assisted EHR systems have become popular for outsourcing medical record management to the CSP and reducing management overhead. However, concerns about security and privacy arise when storing sensitive medical record data on cloud servers. Additionally, traditional encryption methods cannot achieve fine-grained data sharing and access. Access to such data without authorization can result in serious consequences, including identity theft, insurance fraud, and even the endangering of patient lives. Therefore, it is crucial to design robust security mechanisms for protecting medical data stored in the cloud [3]. To ensure data confidentiality and fine-grained access control to sensitive EHR data, Attribute-Based Encryption (ABE) was introduced as an appropriate encryption solution [4].

CP-ABE, proposed by Bethencourt et al. [5], provides a flexible way for data owners to specify their own access control policies. This is achieved by linking a ciphertext to an access policy defined by the user and associating a user’s secret key with a set of attributes. As a result, only users whose attributes satisfy the access policy can decrypt the ciphertext and access the data. However, existing CP-ABE schemes [5,6,7] may not meet security and privacy requirements. Firstly, the access policy that is associated with the ciphertext can be viewed publicly, which means that anyone can obtain useful sensitive private data and expose confidential details regarding the encrypted EHR data, which can result in the exposure of the identity and attributes of medical practitioners or patients, thus compromising their privacy [8]. In addition, those existing schemes have the disadvantage of having a linear increase in ciphertext size and decryption overhead with the number of attributes in the access policy, which can be a significant limitation for resource-limited users [9]. As more attributes are added to the policy, both the ciphertext size and decryption overhead grow proportionally. This can make it difficult or even impossible for users with limited resources to decrypt and access the data that they need in a timely manner. Furthermore, this linear growth in ciphertext size can increase the cost of storing and transmitting the encrypted data, as well as the computational overhead associated with performing encryption and decryption operations. Thus, these CP-ABE schemes may not be suitable for applications that require efficient access control and low computational overhead.

Some existing works have been proposed to address these issues, such as outsourcing decryption technology [9,10,11,12] to reduce computation overhead for data users or proposing fully or partially hidden access structure schemes [8,13,14] to protect the privacy of user attributes embedded in the access policy. However, these solutions fail to offer a large universe and reliable mechanism with adaptive security, or they lack flexibility and have low efficiency. To tackle these issues, we propose an efficient access control scheme with robust privacy protection and outsourced decryption with verifiability, which builds upon the work [15] with further optimizations. Our main contributions are outlined as follows:

- We designed an efficient and secure fine-grained access control scheme named EVOAC-HP for privacy preserving in the medical data scenario, which supports a large universe, adaptive security and flexible access control structure. This scheme uses the attribute bloom filter to achieve policy hiding, which effectively protects data users’ attribute information.
- We adopted outsourcing decryption technology to outsource complex computation to the CSP and support the verification of the outsourcing results, which largely reduces the computation overhead of data users.
- According to security analysis and performance analysis, EVOAC-HP demonstrated enhanced security and efficiency, which is appropriate for medical data-sharing application scenarios with high privacy requirements.

## 2. Related Work

Traditional identity-based encryption (IBE) schemes associate encryption keys with specific identities, and decryption is only possible for ciphertexts encrypted under the matching identities [16]. In contrast, fuzzy IBE allows partial matches between the encryption key and the access policy to be performed, which means that ciphertexts can be decrypted by users whose identities only partially match the policy. In order to achieve more fine-grained access control, the notion of ABE, which enables secure encryption and decryption to be achieved without the need for exact identity matching, was first conceived by Sahai and Waters [4]. According to different application scenarios, ABE is divided into Key-Policy Attribute-Based Encryption (KP-ABE) and CP-ABE. In 2006, Goyal et al. [17] proposed the first construction of a scheme that associates attributes with the private keys of the users, mainly used in paid video sites and broadcast encryption. The first CP-ABE scheme proposed by the work [5] allows complex access policies in which attributes are associated with the ciphertext to be used, which is suitable for data sharing. Although this scheme is very expressive, it was proved to be safe under the general group model, and the security of this model is relatively weak. Afterwards, a significant amount of research has been conducted on CP-ABE and KP-ABE in recent years, with various constructions and optimizations proposed to enhance their efficiency and security. Waters [18] proposed an improvement to CP-ABE schemes by introducing a linear secret sharing scheme (LSSS) based on the previous work [5], which results in enhanced efficiency and expressiveness of the encryption scheme while maintaining stronger security. Many CP-ABE schemes have since emerged, including some classic works [6,7,15]. However, as the access policy becomes more complex and the number of user attributes increases, the size of the ciphertext and the computational overhead required for decryption also increase. Since the decryption process can be expensive due to the large size of the ciphertext and the required pairing operations, resource-limited devices may experience significant delay or even have to abort the decryption process.

To address this inefficiency issue, Green et al. [9] introduced the innovative notion of ABE with outsourced decryption (ABE-OD), which allows a CSP to transform any ABE ciphertext of any size into an ElGamal-type ciphertext with a constant size, so that the user only needs to obtain the decrypted message with one exponentiation. One limitation of outsourcing decryption to the CSP is that they may not perform the transformation operations honestly. To address this concern, the work [10] proposed the concept of ABE verifiable outsourced decryption (ABE-VOD), which enables data users to check whether the transformation results are correct. However, this scheme needs to encrypt additional random messages, which doubles the computing overhead of the data user, resulting in twice the size of the ciphertext. This undoubtedly increases the user’s bandwidth requirements and computing costs. Afterward, several efficient and generic ABE-VOD schemes have been proposed [11,19,20,21,22,23,24]. Some works [19,20] also considered performing outsourced key issuing and outsourced decryption operations by introducing two cloud service providers. Mao et al. [11] and Lin et al. [21] proposed an efficient construction using a commitment scheme and an encapsulation mechanism. These ABE schemes have shown great promise in enabling the secure and verifiable outsourced decryption of ciphertexts to be performed in cloud-based environments. While these ABE schemes are well suited to flexible and fine-grained data-sharing systems, they may not be suitable for applications with high privacy requirements, such as EHR systems. This is because the access policy, which typically includes sensitive attribute information, such as the patient’s medical condition and medical history, is directly exposed in the ciphertext. Anyone can obtain some sensitive information with the ciphertext. As a result, malicious adversaries are likely to infer sensitive information about the patient’s health status, potentially leading to privacy breaches and other security risks.

In order to improve the above privacy protection issues, CP-ABE with policy hiding has been researched to address sensitive attribute information exposure in the access policy of traditional CP-ABE schemes. The concept of partially hidden access structures was proposed by Nishide et al. [8] and splits the attribute into an attribute name exposed to the ciphertext and sensitive attribute values hidden within the ciphertext. Nevertheless, this solution only supports the less expressive “AND gates” access structure. Lai et al. [25] proposed a CP-ABE scheme with adaptive security and policy hiding under the standard model, but this scheme is constructed based on bilinear groups of composite order, resulting in low efficiency. Subsequently, several works based on [8,25] have since been proposed to enhance the security, efficiency, flexibility and availability of partially hidden access structure CP-ABE [14,26,27,28,29,30]. However, all these existing schemes can only hide the attribute value but cannot hide the attribute name, and the attribute name itself still leaks some useful information. Therefore, CP-ABE schemes with fully hidden policy are also available, where the access policy is invisible to the data user, ensuring that their attribute sets are not revealed in plaintext. Recently, most of the solutions hide the whole attribute by employing the specified garbled bloom filter [31] to eliminate the attribute mapping function embedded in the access structure and achieve full policy hiding [32,33,34,35,36]. This strategy of policy hiding is well suited to applications with strong privacy requirements. However, these schemes either do not support verifiable outsourced decryption or do not support flexible access structures under the standard model.

Inspired by the works [11,15,32], we propose EVOAC-HP to be applied in EHR systems. Consider a healthcare scenario where a data owner, such as a patient, wants to share their medical records with a data user, such as a doctor, but does not want to allow other doctors or unauthorized users to access the records. To protect privacy, the patient first establishes an access policy for who is allowed to access the records and encrypts the records using CP-ABE before uploading them to a CSP. Then, doctors who meet the defined access policy can successfully decrypt the ciphertext. Note that the private attributes embedded in the access policy do not reveal the identity of the doctors. Since the doctor’s device may be resource-limited, they can request the CSP to transform the ciphertext and obtain the transformed ciphertext for decryption. Finally, the doctor can locally confirm whether the transformed ciphertext is correct to ensure the integrity of the decryption result.

## 3. Preliminary

In this section, we mainly review the fundamental knowledge about the CP-ABE definition, the security model for the CP-ABE, the commitment scheme, the attribute bloom filter and the decisional linear assumption.

### 3.1. CP-ABE Definition

A classic CP-ABE scheme is given by the following four algorithms:

- $Setup(1^{\lambda },U)$: This algorithm takes the security parameter λ and attribute universe description *U* as input, and it outputs a master public key mpk and a master key msk.
- KeyGen(msk,S): This algorithm takes the master key msk and a set of attributes *S*, and it outputs a secret decryption key sk.
- Encrypt(mpk,(M,ρ),M): This algorithm takes the master public key mpk, an access structure (M,ρ) over the universe of attributes and a message *M*, and it outputs a ciphertext ct.
- Decrypt(mpk,ct,sk): This algorithm takes the master public key mpk, a ciphertext ct and a secret decryption key sk, and it outputs a message *M* or a special symbol ⊥.

### 3.2. Security Model for CP-ABE

We introduce the security model for the CP-ABE scheme. The security between adversary A and challenger C is given as follows:

- Initialization: A defines the challenge access structure $A^{*}$ and gives it to C.
- Setup: C runs Setup to obtain the master public key mpk and returns it to A.
- Phase1: A has the ability to request secret keys corresponding to collections of user attributes $S_{1},S_{2}$, $\ldots ,S_{Q_{1}}$. For each $S_{i}$, C runs KeyGen and returns $sk_{i}$ to A. The queried sets must not meet the access structure requirements of the challenge phase above each query, i.e., $\forall i\in \left[Q_{1}\right]:S_{i}\notin$$A^{*}$.
- Challenge: A submits two messages, $m_{0}$ and $m_{1}$, with equal length and returns them to C. C chooses random number b∈{0,1} and invokes Encrypt to obtain ct, which is given to A.
- Phase2: Phase 1 is reiterated under the same constraint. A can continue to query the secret keys for the sets $S_{Q_{1}+1},S_{Q_{1}+2}$, $\ldots ,S_{Q}$.
- Guess: A outputs his guess value $b^{\prime }\in \left\{0,1\right\}$ for *b*.

**Definition 1.**  *A CP-ABE scheme* Π *is secure if all polynomial-time attackers A have at most a negligible advantage in the above security game. The advantage of an attacker A for breaking this confidentiality game is defined as $Adv_{\Pi }^{A}\left(\lambda \right)=\left|Pr\left[b^{\prime }=b\right]-1/2\right|$.*

### 3.3. Commitment

A commitment scheme has three algorithms: InitCom, Commit and Reveal.

- $Setup(1^{\lambda })$: This algorithm takes a security parameter λ as input and outputs the public commitment key pk.
- Commit(M,r): This algorithm takes a message *M* and an additional random number *r* as input and outputs a commitment cm.
- Reveal(M,r,cm): This algorithm takes a message *M*, an additional random number *r* and a commitment cm as input and outputs a bit value b∈{0,1}. If b=1, it indicates that cm is a valid commitment to *M*; otherwise, it indicates that cm is not a commitment to *M*.

A correct commitment scheme satisfies Reveal(M,r,Commit(M,r))=1 for all pk output by the algorithm Setup and for any message *M* and an additional random number *r*. If a commitment scheme satisfies the hiding property and the binding property, it is considered secure. The commitment cm to *M* satisfies the hiding property, which ensures that no information about *M* can be inferred from cm. The binding property ensures that it is computationally infeasible to reveal a commitment cm to a value $M_{EHR}^{\prime }$ other than the original value *M*.

### 3.4. Attribute Bloom Filter

The attribute bloom filter (ABF) is a data structure that can efficiently query a large dataset [32]. It is a variant of the bloom filter, where each element is assigned one or more attributes so that it can be filtered and queried by the attribute. During the search stage, one or more attributes can be specified, and only elements with the specified attributes are returned. Unlike traditional bloom filters, the ABF stores an additional attribute vector, where each element corresponds to an attribute vector. Each position in the attribute vector corresponds to an attribute, and if the element has that attribute, the position is set to 1; otherwise, 0. The element is returned during the query stage if all specified attribute positions are 1.

To construct the ABF, a sequence of λ bits in length is joined with two constant strings: one that includes the row number with $L_{rownum}$ bits and another that contains the attribute with $L_{att}$ bits, where the security parameter $\lambda =L_{rownum}+L_{attr}$. The ABF can be divided into two components: ABFBuild and ABFQuery. These two components are detailed below:

- ABFBulid(M,ρ): This algorithm takes as input an access policy (M,ρ). The attributes $att_{e}$ and their corresponding row number *i* involved in access matrix M are concatenated, where each row *i* maps to attribute $att_{e}$ according to the mapping function ρ(i), and they are left-padded with zeros to achieve a maximum bit length. The resulting set of elements $S_{et}={\left\{i||att_{et}\right\}}_{i\in [1,\ldots ,l]}$ is used as input in the garbled BF building algorithm to create $ABF_{S}$, as described in [31].
  When an element et needs to be inserted into $ABF_{S}$, this algorithm randomly produces x−1λ-bit strings $r_{1},r_{2},\cdots ,r_{x-1}$ and adopts the XOR-based secret sharing scheme (x,x) to distribute element et. It then assigns $r_{x}=r_{1}⊕r_{2}⊕\cdots ⊕r_{x-1}⊕et$. Afterward, *x* independent and uniform hash functions $H_{i}$ are used to hash attribute $att_{e}$ to obtain $H_{i}\left(att_{et}\right)$ for i∈[1,…,x], where $H_{i}\left(att_{et}\right)$ represents the specific index position in $ABF_{S}$. The *i*-th share $r_{i}$ of the element is then stored in $ABF_{S}$ at the position indicated by the hash function $H_{i}\left(att_{et}\right)$. When adding more elements to $ABF_{S}$, it is possible that a location $j=H_{i}\left(et\right)$ has already been filled by an element added earlier. In this case, we can repurpose the existing share assigned as a share for the new element.
- $ABFQuery(mpk,S,ABF_{S})$: This algorithm takes as input the master public key mpk, an attribute set *S* and the attribute bloom filter $ABF_{S}$. For each attribute att within the attribute set *S*, the algorithm calculates the position index $H_{i}\left(att_{et}\right)$ in $ABF_{S}$ with *x* hash functions. The value $r_{i}$ stored in the corresponding position $H_{i}\left(att\right)$ can then be retrieved.
  Once the element shares the $r_{i}$ that have been stored in $ABF_{S}$, element et can be reconstructed using the formula $et=r_{1}⊕r_{2}⊕\ldots ⊕r_{x-1}⊕r_{k}=r_{1}⊕r_{2}⊕\ldots ⊕r_{x-1}⊕r_{k}⊕et$, where et is represented as $i||att_{e}$ and attribute $att_{e}$ is derived from the rightmost $L_{att}$ bits of element et. If there are redundant zeros in $att_{e}$, they are removed. When $att_{e}$ matches the attribute att specified in the access policy, this implies that attribute att exists in the access control policy. Otherwise, attribute att is not present in the access policy. Similarly, the row number *i* is derived from the leftmost *L* bits of element et, and redundant zeros are removed if present. Once $att_{e}$ and *i* have been obtained, the attribute mapping can be rebuilt as $\rho^{\prime }={\left(rownum,att\right)}_{att\in S}$.

Above all, the ABFBuild algorithm hides the attribute mapping, while the ABFQuery algorithm reconstructs it. This access control structure can allow full policy hiding to be achieved.

### 3.5. Decisional Linear Assumption

Suppose we have two multiplicative groups, G and H, of prime number order *q* and two generators, g∈G and h∈H. We randomly select $x,y,m,n\in Z_{p}$ and define a tuple $T_{1}=\left(g,h,g^{x},g^{y},h^{x},h^{y},g^{mx},g^{ny},h^{mx},h^{ny},g^{m+n},h^{m+n}\right)$. We randomly choose a random number $\mu \in Z_{p}$ and define another tuple $T_{2}=\left(g,h,g^{x},g^{y},h^{x},h^{y},g^{mx},g^{ny},h^{mx},h^{ny},g^{\mu },h^{\mu }\right)$. The assumption then is that no polynomial-time adversary can distinguish tuple $T_{1}$ from tuple $T_{2}$ with negligible advantage.

## 4. Access Control System

In this section, we introduce our system model and the concrete EVOAC-HP, which is composed of five stages, including system parameter initialization, key generation, EHR data encryption, ciphertext transformation, and EHR data decryption and verification.

### 4.1. System Model

**Figure 1:**
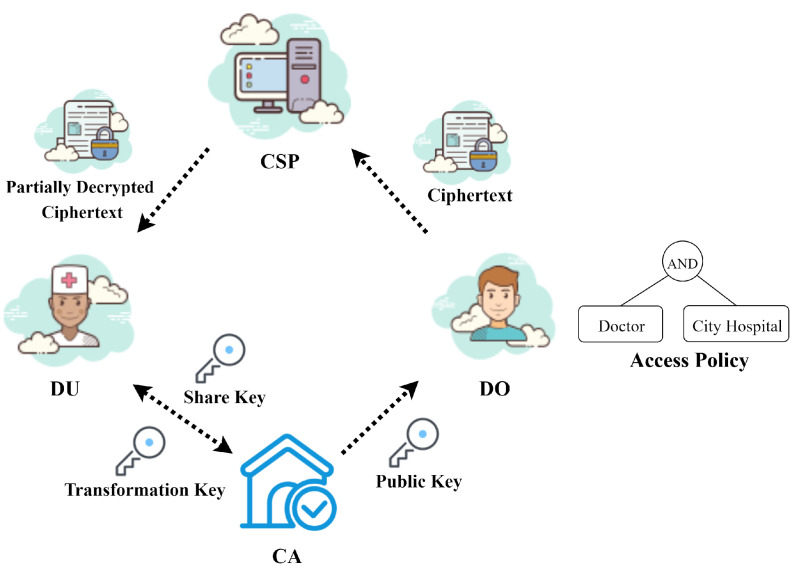
The system model of EVOAC-HP.

As shown in Figure 1, the system model of EVOAC-HP comprises four entities: a certificate authority (CA), many data owners (DOs), many data users (DUs) and a cloud service provider (CSP). The following provides the definition and functions of each participant:

- **Certificate authority (CA):** The central component of the entire system is the CA, which is in charge of the initialization of the system. The CA is responsible for creating the master private key and public parameters. Additionally, the CA generates transformation keys for users based on their attribute set when requested. It is assumed that the CA is a trusted entity.
- **Data owners (DOs):** DOs are the producers of medical data. When they need to share their data, they first establish a custom access policy that allows users to access and encrypt the data using our proposed encryption scheme. The resulting ciphertext is then uploaded to the CSP. It is worth noting that the access policy in the ciphertext does not reveal the sensitive attributes of the data owner. In a medical scenario, the data owners are the patients.
- **Data users (DUs):** In the healthcare scenario, the DUs play the role of a doctor or a nurse. They need to retrieve the corresponding ciphertext from the CSP and request it to assist in the transformation of the ciphertext. When obtaining the transformed decrypted ciphertext from the CSP, the data user locally decrypts it to access the EHR data and checks whether the transformation result is correct. Considering that the data user is likely to use resource-limited devices such as conventional desktop computers, the decryption operation is delegated to simplify the computation.
- **Cloud service provider (CSP):** The CSP is a powerful server with storage and computing services. Its main responsibilities include storing ciphertext corresponding to medical data and providing ciphertext transformation service. It should be noted that the CSP is honest but curious, which means that it may attempt to extract useful information from the ciphertext.

### 4.2. The Proposed Scheme

**System parameter initialization.** The entire process of system initialization is performed by the CA. It runs $Setup(1^{\lambda },U)$ to generate (mpk,msk). This process is shown in Algorithm 1. This algorithm takes as input the security parameter λ and a large attribute universe description *U*, and it outputs the master public key mpk and the master key msk. In this process, the CA executes the initialization stage of the FAME scheme [15] and the commitment scheme. In addition, the attribute bloom filter is parameterized. Finally, the master public key is mpk, and the master key is msk.
**Algorithm 1** Setup (Invoked by CA).**Input** Security parameter λ, attribute universe description *U*

**Output:** The master public key mpk, the master key msk

1:Suppose $L_{att}$ and $L_{rownum}$ denote the maximum bit length of attributes in the whole system and the row numbers in the access matrix. The ABF has a bit array of size $L_{ABF}$ and is associated with *x* hash functions;2:Generate *x* hash functions $H_{i}$ for i∈[1,…,x]: $H_{i}:{\left\{0,1\right\}}^{*}\to \left[1,L_{ABF}\right]$ and a hash function $H:{\left\{0,1\right\}}^{*}\to G;$3:Randomly pick three groups G, H and $G_{T}$ of prime order *p* and defines a bilinear map $\hat{e}:G\times H\to G_{T}$ and generators $g_{0},g,h_{0}\in G,h\in H$ and random number $a_{1},a_{2},b_{1},b_{2}\in Z_{p}^{*}$ and $d_{1},d_{2},d_{3}\in Z_{p};$4:Calculate $A_{1}=h^{a_{1}},A_{2}=h^{a_{2}},E_{1}=e{\left(g,h\right)}^{d_{1}a_{1}+d_{3}},E_{2}=e{\left(g,h\right)}^{d_{2}a_{2}+d_{3}};$5:Set the master public key $mpk=\{g_{0},h_{0},h,A_{1},A_{2},E_{1},E_{2},H_{i\in [1,\ldots ,x]},H\};$6:Set the master key $msk=\{g,h,a_{1},a_{2},b_{1},b_{2},g^{d_{1}},g^{d_{2}},g^{d_{3}}\}.$


**Key generation.** This stage has two steps: Firstly, the data user randomly selects $z\in Z_{p}$ as a secret decryption key dk=z and produces a shared public key $spk=g^{\frac{1}{z}}$. When the data user needs to apply for the transformation key, the data user sends the shared public key spk to the CA responsible for issuing transformation keys. The CA runs TkGen(msk,S,spk) to generate the transformation key tk, and this process is shown in Algorithm 2. This algorithm takes as input the master key msk, a set of the data user attributes *S* and a shared public key spk, and it outputs the transformation key $tk=(tk_{0},{\left\{tk_{y}\right\}}_{y\in S},tk^{\prime }).$
**Algorithm 2** TkGen (Invoked by CA).**Input** The master key msk, attribute set *S*, a shared public key spk

**Output:** Transformation key tk

1:Randomly chooses two random number $r_{1},r_{2}\in Z_{p}$ and calculate $tk_{0,1}=h^{b_{1}r_{1}},tk_{0,2}=h^{b_{2}r_{2}},tk_{0,3}=h^{r_{1}+r_{2}};$2:Set $tk_{0}=\left(tk_{0,1},tk_{0,2},tk_{0,3}\right);$3:**for** each y∈S **do**4:   **for** k∈{1,2} **do**5:     Randomly chooses a random number $\sigma_{y}\in Z_{p}$ and calculates $tk_{y,k}=H{\left(y1k\right)}^{\frac{b_{1}r_{1}}{a_{k}}}\cdot H{\left(y2k\right)}^{\frac{b_{2}r_{2}}{a_{k}}}\cdot$$H{\left(y3k\right)}^{\frac{r_{1}+r_{2}}{a_{k}}}\cdot g^{\frac{\sigma_{y}}{a_{k}}}$;6:   **end for**7:**end for**8:Set $tk_{y}=\left(tk_{y,1},tk_{y,2},g^{-\sigma_{y}}\right);$9:**for** k∈{1,2} **do**10:   Randomly chooses $\sigma^{\prime }\in Z_{p}$ and calculates $tk_{k}^{\prime }=g^{\frac{d_{k}}{z}}\cdot H{\left(011t\right)}^{\frac{b_{1}r_{1}}{a_{k}}}.H{\left(012t\right)}^{\frac{b_{2}r_{2}}{a_{k}}}\cdot H{\left(013t\right)}^{\frac{r_{1}+r_{2}}{a_{k}}}\cdot g^{\frac{\sigma^{\prime }}{a_{k}}};$11:**end for**12:Set $tk^{\prime }=\left(tk_{1}^{\prime },tk_{2}^{\prime },g^{\frac{d_{3}}{z}}\cdot g^{-\sigma^{\prime }}\right);$13:Set $tk=(tk_{0},{\left\{tk_{y}\right\}}_{y\in S},tk^{\prime }).$


**EHR data encryption.** When encrypting sensitive EHR data $M_{EHR}$ under the defined access structure (M,ρ), the data owner first randomly picks a value *r* and uses a concatenation operation represented by “||” to encrypt the combination of EHR data and the random number $M_{EHR}\left|\right|r$. This means that the encrypted result contains both the original EHR data and the random value. That is, it runs $Encrypt(mpk,M_{EHR}||r,\left(M,\rho \right))\to ct$. This algorithm takes the master public key mpk, the EHR data $M_{EHR}$, the random number *r* and the access structure (M,ρ) as input, and it outputs the encrypted EHR data ct. This process is shown in Algorithm 3. To hide the access structure (M,ρ), we adopt the attribute bloom filter to achieve this property. It invokes $ABFBulid\left(M,\rho \right)\to ABF_{S}$ to obtain $ABF_{S}$. This algorithm takes as input the access structure (M,ρ), and it outputs the attribute bloom filter $ABF_{S}$. The concrete process is detailed in Section 3.5. In order to commit to $M_{EHR}$ using the value *r*, it runs $Commit(M_{EHR},r)\to cm$. The data owner calculates a commitment of the EHR data $cm=g_{0}^{M_{EHR}}h_{0}^{r}$. The final ciphertext $ct^{\prime }$ is $\left(ct,cm,M,ABF_{S}\right)$.
**Algorithm 3** Encrypt (Invoked by DO).**Input** Master public key mpk, the combination of the message and random number $\left(M_{EHR}||r\right)$, access structure (M,ρ), attribute set *S*

**Output:** Encrypted message ct

1:Randomly chooses two random number $s_{1},s_{2}\in Z_{p}$ and calculates $ct_{0,1}=A_{1}^{s_{1}},ct_{0,2}=A_{2}^{s_{2}},ct_{0,3}=h^{s_{1}+s_{2}};$2:Set $ct_{0}=\left(ct_{0,1},ct_{0,2},ct_{0,3}\right);$3:**for** each $i\in (1,n_{1})$ **do**4:   **for** l∈{1,2,3} **do**5:     Calculate $ct_{i,l}=H{\left(\rho \left(i\right)l1\right)}^{s_{1}}\cdot H{\left(\rho \left(i\right)l2\right)}^{s_{2}}\cdot \prod_{j=1}^{n_{2}}{\left[H{\left(0jl1\right)}^{s_{1}}\cdot H{\left(0jl2\right)}^{s_{2}}\right]}^{{\left(M\right)}_{i,j}};$6:   **end for**7:**end for**8:Set $ct_{i}=\left(ct_{i,1},ct_{i,2},ct_{i,3}\right);$9:Calculate $ct_{M_{EHR}}=E_{1}^{s_{1}}\cdot E_{2}^{s_{2}}\cdot (M_{EHR}||r);$10:Set $ct=(ct_{0},ct_{1},\cdots ,ct_{n_{1}},ct_{M_{EHR}}).$


**Ciphertext transformation.** In this stage, the CSP invokes Transform(ct,tk)→pct. This algorithm takes as input the encrypted EHR data ct and the transformation key tk and outputs the partially decrypted ciphertext pct. This process is shown in Algorithm 4. Notice that the CSP might be malicious or lazy, so the transformation result is not necessarily true.
**Algorithm 4** Transform (Invoked by CSP).**Input** Ciphertext ct, transformation key tk

**Output:** Partially-decrypted ciphertext pct

1:Calculate $num=e\left(\prod_{i\in I}^{\gamma_{i}}ct_{i,1}^{\gamma_{i}},tk_{0,1}\right)\cdot e\left(\prod_{i\in I}^{\gamma_{i}}ct_{i,2}^{\gamma_{i}},tk_{0,2}\right)\cdot e\left(\prod_{i\in I}^{\gamma_{i}}ct_{i,3}^{\gamma_{i}},tk_{0,3}\right);$2:Calculate $den=e\left(tk_{1}^{\prime }\cdot \prod_{i\in I}tk_{\rho (i),1}^{\gamma_{i}},ct_{0,1}\right)\cdot e\left(tk_{2}^{\prime }\cdot \prod_{i\in I}tk_{\rho (i),2}^{\gamma_{i}},ct_{0,2}\right)\cdot e\left(tk_{3}^{\prime }\cdot \prod_{i\in I}tk_{\rho (i),3}^{\gamma_{i}},ct_{0,3}\right);$3:Calculate pct=num/den.


**EHR data decryption and verification.** When the data user requests the full ciphertext from the CSP, it firstly decomposes the ciphertext $ct^{\prime }$ into $\left(ct,cm,M,ABF_{S}\right)$ and runs $ABFQuery\left(mpk,S,ABF_{S}\right)\to \rho^{\prime }$. The concrete process is detailed in Section 3.5. This algorithm takes as input the master public key mpk, a set of data user attributes *S* and attribute bloom filter $ABF_{S}$ and outputs the rebuilt attribute mapping $\rho^{\prime }$. When recovering the corresponding access structure $\left(M,\rho^{\prime }\right)$, the resource-constrained data user is able to delegate the ciphertext ct to the CSP for decryption; the CSP transforms the ciphertext ct into partially decrypted ciphertext pct and gives it to the data user. Then, the data user runs $Decrpt(ct^{\prime },pct,dk)$. This algorithm takes as input the full ciphertext $ct^{\prime }$, the partially decrypted ciphertext pct and decryption key dk=z and outputs the final result $result=ct_{M_{EHR}}\cdot pct^{z}$. The result is the combination of the message and the random number $\left(M_{EHR}||r\right)$. Aiming to check the correctness of the transformation result, the data user runs $Reveal(M_{EHR},r,cm)$. This process checks whether $cm\overset{\text{?}}{=}g_{0}^{M_{EHR}}h_{0}^{r}$. If it passes verification, the data user finally obtains the true message, $M_{EHR}$. Otherwise, the transformation result is wrong.

## 5. Security Analysis

**Theorem 1.**  *EVOAC-HP can guarantee the confidentiality of data under the random oracle model.*

**Lemma 1.**  *If FAME [15] can guarantee the confidentiality of data, the proposed scheme can also guarantee the confidentiality of data.*

**Proof.**  Suppose that there is an adversary that solves the difficult decisional linear problem in probabilistic polynomial time, whose advantage ε cannot be ignored. With the help of adversary A, an algorithm Alg can be constructed so that the algorithm can break the confidentiality of FAME in probabilistic polynomial time, and the advantages are not negligible.

Let C be the challenger of a safe game in the FAME scheme, and let A be the adversary of the game. Algorithm Alg can break FAME’s confidentiality by performing the following:

- System initialization: Adversary A sends the access structure $A^{*}$ that needs to be challenged to algorithm Alg. Algorithm Alg gives $A^{*}$ to challenger C. Challenger C generates public parameter $mpk=\{h,A_{1},A_{2},E_{1},E_{2},H\}$ with the Setup algorithm in FAME and sends mpk to algorithm Alg. Algorithm Alg calls the Setup algorithm in EVOAC-HP to complete public parameter $mpk=\{g_{0},h_{0},h,A_{1},A_{2},E_{1},E_{2},H_{i\in [1,\ldots ,x]},H\}$ and send it to adversary A.
- Query phase 1: Algorithm Alg receives the decryption key query request submitted by adversary A. Let adversary A ask for the decryption key of the attribute set $S_{e}$, and let algorithm Alg forward this query request to challenger C. Challenger C calls the KeyGen algorithm in the FAME scheme to output the decryption key given to algorithm Alg, and algorithm Alg forwards its key to adversary A.
- Challenge phase: Adversary A submits two equal-length plaintexts, $m_{0}^{*}$ and $m_{1}^{*}$, to algorithm Alg. Algorithm Alg sends them to challenger C and asks for the challenge ciphertext. Challenger C randomly selects b∈{0,1} and calls the Encrypt algorithm in the FAME scheme to encrypt the message $m_{b}^{*}$. Finally, challenger C sends the ciphertext ct to algorithm Alg. After receiving the challenge ciphertext ct, algorithm Alg completes ct according to the ciphertext form of EVOAC-HP and then sends it to adversary A.
- Query phase 2: This phase is the same as Query phase 1.
- Guess phase: If adversary A outputs a bit $b^{\prime }$, then algorithm Alg also outputs $b^{\prime }$.

If adversary A can break the FAME scheme, it means that adversary A can calculate the ciphertext $e{\left(g,h\right)}^{-E_{1}^{s_{1}}\cdot E_{2}^{s_{2}}}$ according to algorithm Alg. With the help of adversary A, algorithm Alg can calculate $m_{b}^{*}$ in the FAME algorithm, thereby breaking the FAME scheme. □

**Theorem 2.**  *If the decisional linear assumption is correct, FAME is adaptively secure under the random oracle model.*

**Lemma 2.**  *FAME [15] has proved that the decisional linear assumption is adaptively secure under the random oracle model. EVOAC-HP is constructed based on FAME, so EVOAC-HP can guarantee the confidentiality of data under the random oracle model.*

**Theorem 3.**  *EVOAC-HP can guarantee the confidentiality of ciphertext transformation.*

**Proof.**  The proposed scheme guarantees transformation result confidentiality with the discrete logarithm problem. Specifically, EVOAC-HP generates a transformation key using a shared public key $spk=g^{\frac{1}{z}}$ provided by the data user. Even if an adversary gains access to the transformation key tk and the intermediate ciphertext ct from the CSP, they cannot compute $ct_{M_{EHR}}\cdot pct^{z}$ without the user’s secret decryption key *z*. Thus, the adversary does not have the ability to decrypt the combination of the EHR data and the random number ($M_{EHR}\left|\right|r$) and gain access to sensitive data $M_{EHR}$. This adds an extra layer of security to the scheme, ensuring that confidential information remains protected even if the adversary gains access to some of the intermediate data. □

**Theorem.** 4 *EVOAC-HP can guarantee the privacy of the access policy.*

**Proof.**  To prevent the potential leakage of attribute information, EVOAC-HP eliminates the attribute mapping function ρ embedded in the access policy using the ABFBulid and ABFQuery procedures. Adversaries without knowledge of the attribute string cannot carry out a successful brute-force attack within polynomial time, so they are incapable of accessing and inferring confidential information from the access policy. Furthermore, data users are only able to check whether they possess the required attributes for accessing the data. It is impossible for a single data user to verify all the attributes from the attribute universe description *U* unless they possess all of the attributes or multiple data users work together to achieve it. Therefore, EVOAC-HP provides protection for policy privacy by concealing attribute information in the access policy. □

## 6. Performance Analysis

In this section, the performance of EVOAC-HP is analyzed from functional, theoretical and experimental perspectives.

### 6.1. Functional Analysis

When it comes to the comparisons of functional analysis, we compare related works in terms of five functionalities: security model, large universe, hidden policy, outsourced decryption and verifiability. Note that in the security model, adaptive security provides stronger security guarantees than selective security. As shown in Table 1, most schemes support only two or three of these features, while the work [18] only supports large universe, and the work [8] only achieves hidden policy. For better security, only schemes [15,25] and EVOAC-HP achieve adaptive security. The schemes [10,11] both support large universe, outsourced decryption and verifiability. According to the above comparison, EVOAC-HP simultaneously achieves large universe, adaptive security, hidden policy, outsourced decryption and verifiability, thus showing functional advantages.

### 6.2. Theoretical Analysis

Here, we only consider some expensive operations and use the symbols Exp, Pair and Hash to denote exponentiation, pairing and hashing operation, respectively. As shown in Table 2, we evaluate EVOAC-HP and compare it with other similar schemes based on the computational complexity of their encryption and decryption algorithms. Regarding the encryption process, although the scheme [32] and EVOAC-HP incur high computational overhead, most of the computational overhead is attributed to the construction of the attribute bloom filter, which requires additional hashing operations to achieve policy hiding. On the other hand, it is obvious that in the decryption stage, existing schemes [15,18,32] need to perform pairing operations, which is expensive for data users, while EVOAC-HP only requires 2Exp+xHash operations. Due to outsourced decryption, EVOAC-HP outperforms other existing schemes that require pairing operations in the decryption stage with regard to performance.

### 6.3. Experimental Analysis

**Figure 2:**
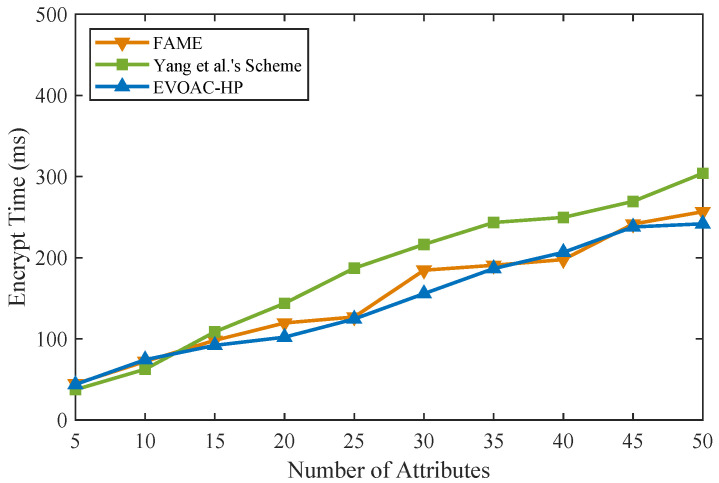
Computation time comparison of data encryption among FAME [15], Yang et al.’s scheme [32] and our scheme.

**Figure 3:**
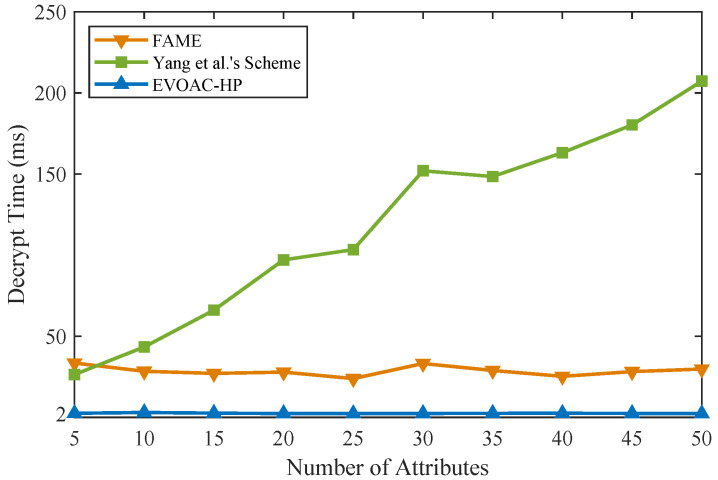
Computation time comparison of data decryption among FAME [15], Yang et al.’s scheme [32] and our scheme.

We implemented similar schemes in Python 3.8 in the same experimental environment (Ubuntu-20.04 with Intel Core i7 and 16 G RAM) on top of the Charm framework [37] and MNT224 curve for pairing. The double-hash technique [38] based on 128-bit MurmurHash and SpookyHash was used to construct *x* hash functions for the attribute bloom filter. We compared the computational time of data encryption and data decryption with schemes [15,32] and EVOAC-HP. We evaluated all the schemes using access policies and attribute sets of sizes ranging from 5 to 50, and the number of hash functions for the ABF was 10. Those schemes were tested in 10 trials, so that the experimental results were averaged. As shown in Figure 2, as the number of attributes increases, the encryption overhead of all three schemes shows a linear growth trend. However, our scheme, EVOAC-HP, has significantly smaller encryption overhead than the scheme proposed in [32], while it has time overhead similar to that of the scheme proposed in [15]. It is worth noting that the scheme proposed in [15] does not consider policy privacy, while our scheme requires the construction of the ABF during the encryption phase to achieve fully hidden policy, resulting in additional computational overhead depending on the number of hash functions used. As shown in Figure 3, the data decryption overhead of scheme [32] shows a linear growth trend as the number of attributes increases, which leads to an increased computational burden for data users. In contrast, EVOAC-HP and scheme [15] maintain constant data decryption overhead. Moreover, EVOAC-HP adopts outsourcing decryption techniques, which further reduces decryption overhead to 2 ms.

**Figure 4:**
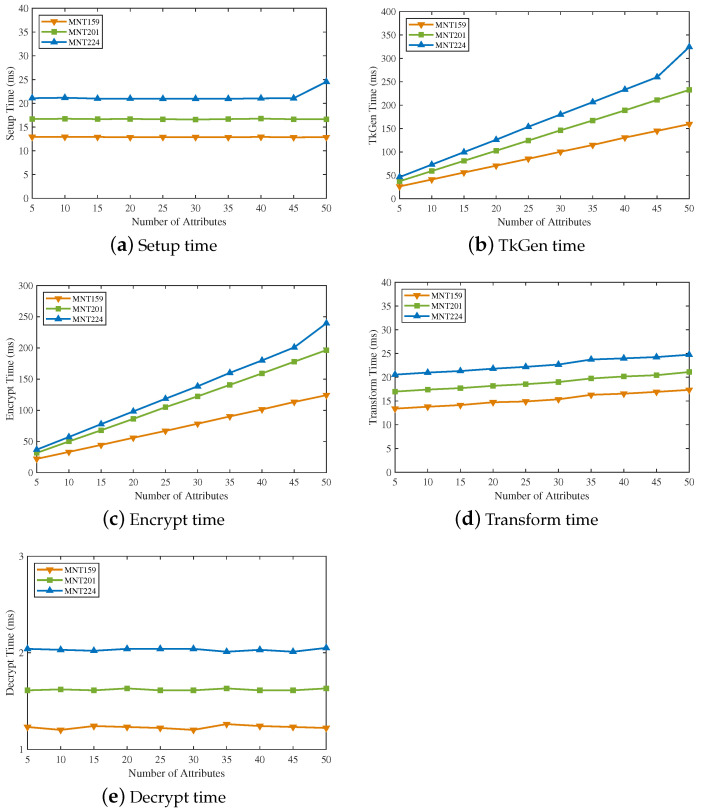
Computational overhead with the number of attributes.

In order to evaluate the time cost of five algorithms in EVOAC-HP, we conducted experiments under three curves, MNT159, MNT201 and MNT224. Figure 4 shows the computational time of each algorithm in our proposed scheme with the number of attributes. Obviously, the MNT224 curve had the highest overhead among all algorithms, while MNT159 had the lowest. The Setup algorithm maintained constant computational overhead, and system initialization was only required once. Except for the Decrypt algorithm, the computation overhead of all algorithms was proportional to the number of attributes. Due to outsourced decryption, the Decrypt algorithm only spent a constant time of around 2 ms.

## 7. Conclusions and Future Work

In this paper, we introduce a practical and reliable scheme for fine-grained privacy protection and outsourced computation access control for sharing medical data, named EVOAC-HP. We employ CP-ABE as a fundamental building block to encrypt the medical data and outsource the decryption operation to alleviate the computation overhead for data users, which reduces the decryption computation overhead to a constant. Additionally, we achieve policy hiding by utilizing the attribute bloom filter, which prevents any individual from accessing sensitive attribute information from the access policy. The user’s attributes are not disclosed in the access policy, which effectively guarantees the security of our scheme. The proposed scheme is proved to be adaptively secure under the random oracle model under the decisional linear assumption. As demonstrated with performance analysis and comparative analysis, EVOAC-HP has functional advantages, better performance and stronger security, which are suitable for EHR data sharing and complex access control in medical scenarios.

Our scheme also has some limitations. First of all, with our scheme, it is difficult to efficiently deal with the policy update problem. When the access policy needs to be updated, the data owner can complete the update with a small computational cost. In this scheme, the data owner needs to re-encrypt according to the new access policy, which is obviously inefficient. Secondly, in most medical scenarios, users’ identities may often change, which requires a new solution that supports user attribute update and revocation to meet this requirement. Finally, our scheme has the problem of offline dictionary attack, that is, the user can continuously query whether the attribute is in the access policy with the attribute bloom filter, which may expose user attributes. In the future, we plan to focus on incorporating features such as CP-ABE with policy update and user attribute revocation while preventing dictionary attacks to better meet practical application scenarios.

## Figures and Tables

**Table 1:** Comparisons of functionality ^1^.

Scheme	Security Model	Large Universe	Hidden Policy	Outsourced Decryption	Verifiability
[8]	Selective security	✘	✔	✘	✘
[18]	Selective security	✔	✘	✘	✘
[9]	Selective security	✔	✘	✔	✘
[25]	Adaptive security	✘	✔	✘	✘
[10]	Selective security	✔	✘	✔	✔
[11]	Selective security	✔	✘	✔	✔
[32]	Selective security	✔	✔	✘	✘
[15]	Adaptive security	✔	✘	✘	✘
EVOAC-HP	Adaptive security	✔	✔	✔	✔

^1^ Here, ✔ denotes support; ✘ denotes no support.

**Table 2:** Comparisons of computation cost ^1^.

Scheme	Encryption	Decryption
[18]	$(2n_{1}+2)Exp$	IExp+(2I+1)Pair
[32]	$(2n_{1}+2)Exp+xHash$	IExp+(2I+1)Pair+xHash
[15]	$\left(6n_{1}+3\right)Exp+6\left(n_{1}+n_{2}\right)Hash$	6Pair
EVOAC-HP	$\left(6n_{1}+3\right)Exp+\left(6n_{1}+6n_{2}+x\right)Hash$	2Exp+xHash

^1^ Here, *I* denotes the number of attributes used in the final successful decryption; $n_{1}$ and $n_{2}$ are the dimensions of the access matrix in the access policy; and *x* is the number of hash functions for the attribute bloom filter.

## Data Availability

Data sharing is not applicable to this article.
